# Manual reduction for incarcerated obturator hernia

**DOI:** 10.1038/s41598-023-31634-4

**Published:** 2023-04-04

**Authors:** Yusuke Gokon, Yusuke Ohki, Takahiro Ogino, Keiichiro Hatoyama, Takahiro Oikawa, Kenji Shimizu, Kazunori Katsura, Takayuki Abe, Koichiro Sato

**Affiliations:** Department of Surgery, Iwate Prefectural Iwai Hospital, 17 Odaira, Kozenji, Ichinoseki, 029-0131 Japan

**Keywords:** Gastrointestinal diseases, Ileum, Jejunum, Surgery

## Abstract

Emergent surgery is a common approach for incarcerated obturator hernias, with high morbidity and mortality rates. Moreover, there have been reports of elective surgery cases after noninvasive manual reduction. For a decade, the initial approach in our institution is also manual reduction unless bowel viability is poor. This study aimed to clarify the efficacy and safety of manual reduction followed by elective surgery. We retrospectively reviewed 50 cases of incarcerated obturator hernia from 2010 to 2022 at Iwate Prefectural Iwai Hospital. Manual reduction was attempted in 31 (62%) patients. The reduction was successful in 21 (42%) patients, and most of them received mesh repair using the extraperitoneal approach as elective surgery. However, two patients underwent emergent surgery in the waiting period because of late-onset constriction and a small bowel perforation. Patients with irreducible hernia underwent emergent surgery, except for two patients who received the best supportive care. Postoperative complications were observed in 5% and 22% of reducible and irreducible cases, respectively. Postoperative mortality was zero in both groups. Manual reduction is useful in some cases, but careful observation is needed because late-onset constriction and perforation could occur.

## Introduction

Obturator hernias (OH) are relatively rare, accounting for 0.07–1% of all hernias^1^. OH occur more frequently in older and slender women^1^. Emergent surgery is a common approach for incarcerated OH, and its morbidity and mortality rates are high because of advanced patient age and serious comorbidities^2^. Moreover, there have been reports of elective surgery after noninvasive manual reduction^3–5^. However, the efficacy and safety of manual reduction are still unclear^5^. For a decade, the initial approach in our institution is also manual reduction unless bowel viability is poor. In this study, we retrospectively reviewed our cases and examined the validity of our approach.

## Methods

This study analyzed 50 patients with incarcerated OH from 2010 to 2022 at Iwate Prefectural Iwai Hospital. All patients were diagnosed with incarcerated OH using computed tomography (CT) scans. We retrospectively reviewed patient characteristics, therapeutic approach, and operative outcomes. Postoperative complications were defined as adverse events occurring within 30 days of surgery or during hospitalization, and severity was assessed using the Clavien–Dindo (C–D) classification (grades I–V)^6^.

The study protocol was approved by our institution's ethics committee or institutional review board (Accession no. Iwai Hospital 2022–1077). Informed consent was obtained from all study participants.

### Manual reduction maneuver

**Figure 1:**
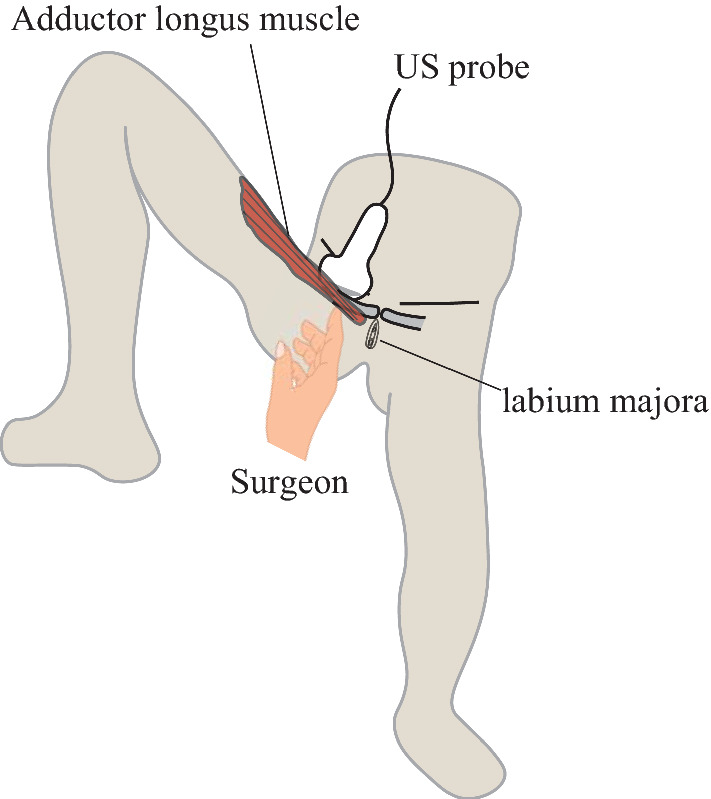
Manual reduction maneuver. The patient is supine with the leg in abduction, flexion, and lateral rotation. The surgeon detected the incarcerated obturator hernia at the anterior side of the adductor longus muscle via ultrasound (US). Manual reduction was performed by compressing the posterior side of the adductor longus muscle and the lateral side of the labium majora.

**Figure 2:**
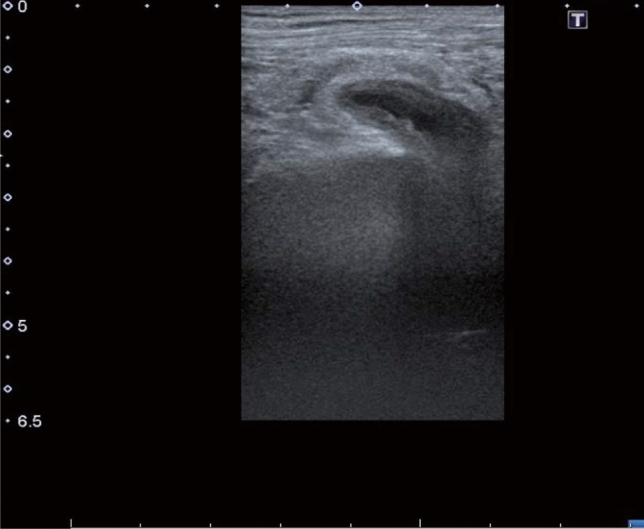
Ultrasound showed the incarcerated small intestine.

**Figure 3:**
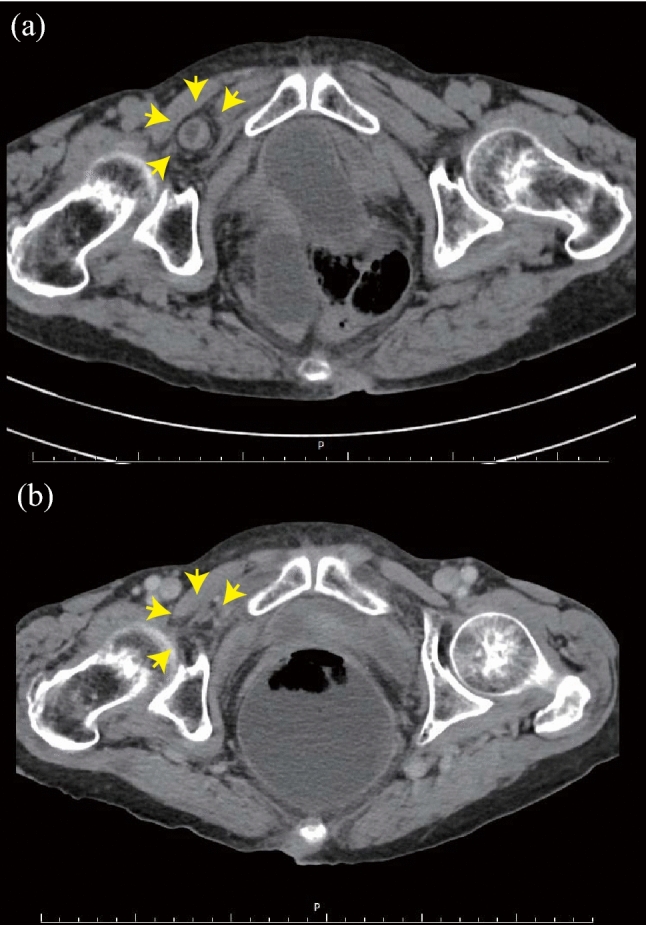
(**a**) Computed tomography showed an incarcerated small intestine (arrows) before manual reduction. (**b**) After reduction, the incarcerated small intestine disappeared (arrows).

The patient was positioned supine with the leg in abduction, flexion, and lateral rotation (Fig. 1). The surgeon visually recognized the incarcerated OH at the anterior side of the adductor longus muscle with ultrasound (Fig. 2). Subsequently, manual reduction was performed by compressing the posterior side of the adductor longus muscle and lateral side of the labium majora manually, until the hypoechoic mass disappeared. If the incarcerated hernia is not released promptly, the assistant bends and stretches the patient’s leg repeatedly^3,4,7^. After the maneuver, the patient underwent a CT scan to confirm the release of the hernia and the absence of perforation (Fig. 3).

### Surgical procedure

**Figure 4:**
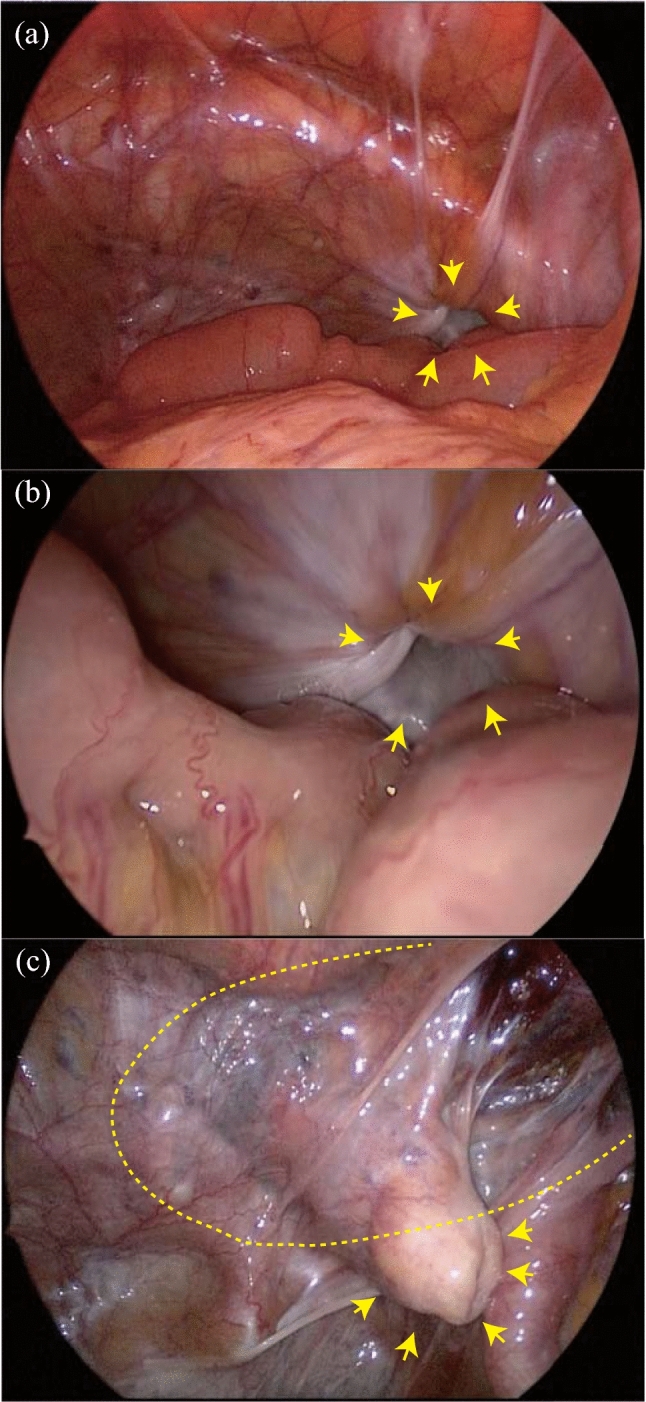
Intraoperative findings. (**a**) Distant and (**b**) close view of obturator hernia (arrows). (**c**) The hernia sac is inverted and not ligated (arrows). BARD ONFLEX modified type L size was placed in the extraperitoneal space (dashed line).

If manual reduction was successful, the suprapubic midline extraperitoneal approach was employed as elective surgery^8^. A 4–6-cm suprapubic vertical incision was made, the space of Retzius was dissected, and the obturator hernia was inverted in direct view. In three extraperitoneal approach cases, the process was visualized laparoscopically (Fig. 4). Conversely, a lower midline laparotomy was employed as emergent surgery.

### Statistical analysis

All statistical analyses were performed using JMP Pro version 15 with Student’s *t* test and Fisher’s exact test. *P* < 0.05 denoted statistical significance.

### Ethical approval and Informed consent

All procedures performed in this study involving human participants were in accordance with the ethical standards of our institutional research committee and with the 1964 Helsinki declaration and its later amendments or comparable ethical standards. Informed consent was obtained from all study participants.

## Results

The patient characteristics are summarized in Table 1. The mean age was 84.8 years. There were 48 female patients and two male patients. The mean interval from symptom onset to hospital admission was 64.9 h. There were 13 American Society of Anesthesiologists physical status (ASA-PS), 2 cases (26%), 33 ASA-PS 3 cases (66%), and 4 ASA-PS 4 cases (8%). Consequently, ASA-PS 4 cases avoided surgery. The overall postoperative morbidity (C–D grade II or higher) rate was 15%: ileus in 11% of the cases, pneumonia in 7%, and tachycardia in 2% (Table 2). Perioperative mortality was zero.

**Table 1:** Patient characteristics. Values in parentheses are percentages unless indicated otherwise † values are mean (SD).

Clinicopathological features	All cases (N = 50)	
Age (years)†	84.8	(7.1)
Gender		
Male	2	(4)
Female	48	(96)
Hernia localization		
Right	29	(58)
Left	21	(42)
Time from the onset (hours)†	64.9	(114)
ASA-PS		
2	13	(26)
3	33	(66)
4	4	(8)

**Table 2:** Postoperative complications. Values in parentheses are percentages.

Postoperative complications*	All cases (N = 46)	
Ileus	5	(11)
Pneumonia	3	(7)
Tachycardia	1	(2)

**Figure 5:**
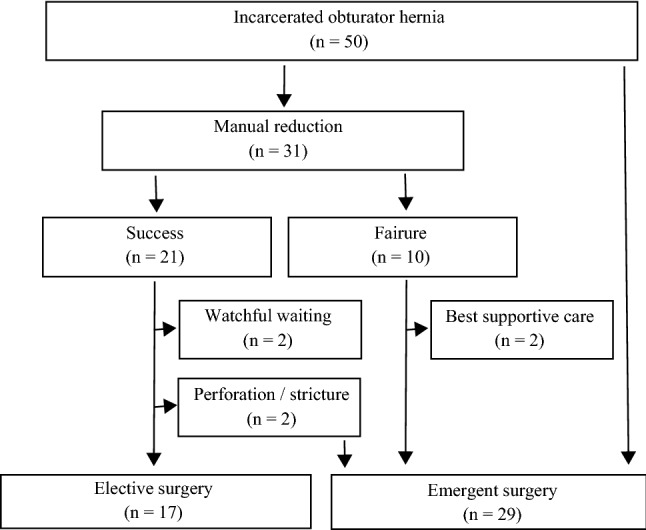
Patient flowchart. Manual reduction was attempted in 31 patients. Reduction was successful in 21 patients, two selected watchful waiting, and 19 selected elective surgeries, but two patients underwent emergent surgery in the waiting period. In 10 patients, manual reduction failed, 8 received emergent surgery, and two received the best supportive care. The remaining 19 patients underwent immediate emergent surgery.

A flowchart of patients is shown in Fig. 5. Manual reduction was attempted in 31 patients. The reduction was successful in 21 patients, of which two selected watchful waiting because of chronic pulmonary insufficiency. The two watchful waiting cases did not involve hernia recurrence, late-onset necrosis, or perforation. One died from acute exacerbation of interstitial lung disease 2 years later, and another died from bacterial pneumonia on hospital day 31. Of the 21 patients, 19 selected elective surgery, but two patients underwent emergent surgery in the waiting period because of late-onset constriction and a small bowel perforation. Manual reduction failed in 10 patients, and 8 underwent emergent surgery. Two patients received the best supportive care because of poor general condition, and they, unfortunately, died the next day. The remaining 19 patients underwent emergent surgery because of suspicions of poor bowel viability.

We divided the patients who underwent manual reduction into two groups: manually reducible and irreducible (Table 3). No significant difference was detected in age, sex, hernia localization, ASA-PS, and laboratory findings between the two groups. The average time from the onset was 39.8 h in the reducible group compared with 73.7 h in the irreducible group (*P* = 0.13).

**Table 3:** Patient characteristics of reducible and irreducible cases. Patients who avoided manual reduction were not included. *Statistical significance. Student's *t* test was used for age, time, and laboratory findings—Fisher’s exact test was used for other parameters. Values in parentheses are percentages unless indicated otherwise † values are mean (SD).

Clinicopathological features	Reducible N = 21 (%)		Irreducible N = 10 (%)		*P* value
Age (years)†	85.7	(7.1)	85.3	(8.6)	0.86
Gender					
Male	0	(0)	1	(10)	0.32
Female	21	(100)	9	(90)	
Hernia localization					
Right	14	(67)	4	(40)	0.25
Left	7	(33)	6	(60)	
Time from the onset (hours)†	39.8	(25.1)	73.7	(66.9)	0.13
ASA-PS					
2	5	(24)	3	(30)	0.62
3	14	(67)	5	(50)	
4	2	(9)	2	(20)	
Laboratory findings					
White blood cell†	9439	(4306)	10,096	(4435)	0.69
C-reactive protein (mg/dL)†	2.4	(4.8)	1.7	(2.2)	0.65
Base excess (mmol/L)†	2.8	(3.9)	1.8	(8.3)	0.67
Lactate (mmol/L)†	1.2	(0.5)	2.6	(4.9)	0.24

We also divided the patients who underwent surgery into manually reducible and irreducible groups (Table 4). The time from arrival at the hospital to surgery was 27 days in the reducible group. In the reducible and irreducible groups, postoperative complications were observed in 5% and 22% of the cases, respectively (*P* = 0.21). Bowel resection was significantly less common in the group that underwent surgery after reduction (*P* = 0.04), whereas mesh repair was significantly common (*P* < 0.001). The group that underwent surgery after reduction included two patients whose elective surgery failed because of constriction and small bowel perforation (Fig. 5).

**Table 4:** Patient characteristics of reducible and irreducible cases. Patients who avoided surgery were not included. *Statistical significance. Student's *t* test was used for age, time, and laboratory findings—Fisher’s exact test was used for other parameters. Values in parentheses are percentages unless indicated otherwise † values are mean (SD).

Clinicopathological features	Reducible N = 19 (%)		Inreducible N = 27 (%)		*P* value
Age (years)†	85.1	(7.0)	84.5	(7.3)	0.78
Gender					
Male	0	(0)	2	(7)	0.50
Female	19	(100)	25	(93)	
The time from arrival at the hospital to surgery (days)†	27	(77.4)	0		0.07
ASA-PS					
2	5	(26)	8	(30)	1.00
3	14	(74)	19	(70)	
4	0	(0)	0	(0)	
Post operative complications					
Absent	18	(95)	21	(78)	0.21
Present	1	(5)	6	(22)	
Bowel resection					
No	17	(89)	16	(59)	0.04*
Yes	2	(11)	11	(41)	
Mesh repair					
No	2	(11)	23	(85)	< 0.001*
Yes	17	(89)	4	(15)	

## Discussion

Our results demonstrated that 21 of 50 (42%) cases of incarcerated OH were reducible at the first visit. When it was reducible, mesh repair was often performed as elective surgery. Although significance was not reached, the complication rate tended to be low in the group that underwent surgery after reduction. A previous report preferred mesh repair to prevent recurrence if there are no contraindications^9^. Moreover, some case reports have suggested that elective surgery after manual reduction is favorable because the surgery can be performed in a favorable condition^3,5^.

These findings suggest the validity of our therapeutic approach, but late-onset constriction and perforation occurred in two patients and needed emergent laparotomy. In inguinal and femoral hernia guidelines, a medical history, systemic inflammatory response syndrome, contrast-enhanced CT findings, lactate, serum creatinine phosphokinase, and D-dimer levels predict bowel strangulation^10^. For example, the time from the onset of ≥ 24 h and lactate level of ≥ 2.0 mmol/L were suggested to be useful predictors of non-viable bowel strangulation^10^. However, a consensus was not established for indications of manual reduction of incarcerated OH^5^. Previous reports have suggested that the criteria for incarcerated OH were less likely to be severe than those for incarcerated inguinal hernias because OH often contains part of the small bowel circumference as a Richter-type hernia^5,11^. In our cases, the mean time from the onset of reducible cases was 40 h, which could be fatal in inguinal hernia^11^. Further investigation is needed to indicate a manual reduction, and careful long-term observation after the manual reduction is needed to make the procedure feasible^3^.

This study has some limitations. First, whether attempting manual reduction or not was based mainly on the surgeon’s discretion. Second, the waiting times for elective surgery were inconsistent.

## Conclusion

Manual reduction followed by elective surgery could be an option for incarcerated OH; however, careful observation after reduction is needed because late-onset constriction and perforation could occur.

## Data Availability

The datasets analyzed during the current study are available from the corresponding author.
